# The Impact of Haplotypes of the *FTO* Gene, Lifestyle, and Dietary Patterns on BMI and Metabolic Syndrome in Polish Young Adult Men

**DOI:** 10.3390/nu16111615

**Published:** 2024-05-25

**Authors:** Sylwia Górczyńska-Kosiorz, Mateusz Lejawa, Marcin Goławski, Agnieszka Tomaszewska, Martyna Fronczek, Beata Maksym, Maciej Banach, Tadeusz Osadnik

**Affiliations:** 1 Department of Internal Medicine, Diabetology and Nephrology, Faculty of Medical Sciences in Zabrze, Medical University of Silesia, 40-055 Katowice, Poland; 2 Department of Pharmacology, Faculty of Medical Sciences in Zabrze, Medical University of Silesia, 40-055 Katowice, Poland; mateusz.lejawa@sum.edu.pl (M.L.); d201182@365.sum.edu.pl (M.G.); mfronczek@sum.edu.pl (M.F.); bmaksym@sum.edu.pl (B.M.); tadeusz.osadnik@sum.edu.pl (T.O.); 3 Prenatal Diagnostic and Genetic Clinic, Medical Center, Medical University of Silesia, 41-800 Zabrze, Poland; atomaszewska@szpital.zabrze.pl; 4 Department of Preventive Cardiology and Lipidology, Medical University of Lodz (MUL), 90-549 Lodz, Poland; maciej.banach@icloud.com; 5 Ciccarone Center for the Prevention of Cardiovascular Disease, Division of Cardiology, Department of Medicine, Johns Hopkins University School of Medicine, 600 N. Wolfe St., Carnegie 591, Baltimore, MD 21287, USA

**Keywords:** *FTO*, haplotypes, gene polymorphism, genetic factors, BMI, metabolic syndrome, diet quality, lifestyle

## Abstract

Background: Variants in fat mass and the obesity-associated protein (*FTO*) gene have long been recognized as the most significant genetic predictors of body fat mass and obesity. Nevertheless, despite the overall evidence, there are conflicting reports regarding the correlation between different polymorphisms of the *FTO* gene and body mass index (BMI). Additionally, it is unclear whether *FTO* influences metabolic syndrome (MetS) through mechanisms other than BMI’s impact. In this work, we aimed to analyze the impact of the following *FTO* polymorphisms on the BMI as well as MetS components in a population of young adult men. Methods: The patient group consisted of 279 Polish young adult men aged 28.92 (4.28) recruited for the MAGNETIC trial. The single-nucleotide polymorphisms (SNPs), located in the first intron of the *FTO* gene, were genotyped, and the results were used to identify “protective” and “risk” haplotypes and diplotypes based on the literature data. Laboratory, as well as anthropometric measurements regarding MetS, were performed. Measured MetS components included those used in the definition in accordance with the current guidelines. Data regarding dietary patterns were also collected, and principal components of the dietary patterns were identified. Results: No statistically significant correlations were identified between the analyzed *FTO* diplotypes and BMI (*p* = 0.53) or other MetS components (waist circumference *p* = 0.55; triglycerides *p* = 0.72; HDL cholesterol *p* = 0.33; blood glucose *p* = 0.20; systolic blood pressure *p* = 0.06; diastolic blood pressure *p* = 0.21). Stratification by the level of physical activity or adherence to the dietary patterns also did not result in any statistically significant result. Conclusions: Some studies have shown that *FTO* SNPs such as rs1421085, rs1121980, rs8050136, rs9939609, and rs9930506 have an impact on the BMI or other MetS components; nevertheless, this was not replicated in this study of Polish young adult males.

## 1. Introduction

Obesity is defined as an excess adipose tissue accumulation. This is often defined as a body mass index (BMI) greater or equal to 30 for adults. In 2016, over 650 million people were obese, and the prevalence of obesity has tripled since 1975. Obesity increases the risk of cardiovascular diseases such as coronary artery disease and stroke, diabetes mellitus (DM), musculoskeletal disorders such as osteoarthritis, and some cancers, including endometrial, breast, ovarian, and prostate cancer [1,2].

Obesity is a component of metabolic syndrome (MetS), an interconnected group of risk factors influencing the development of atherosclerotic cardiovascular disease. Numerous definitions exist [3,4], but one of them was formulated in the Joint Interim Statement of the International Diabetes Federation Task Force on Epidemiology and Prevention; the National Heart, Lung, and Blood Institute; the American Heart Association; the World Heart Federation; the International Atherosclerosis Society; and the International Association for the Study of Obesity. For an adult to be diagnosed with MetS according to this definition, they have to fulfill at least three of the five criteria of abdominal obesity, hypertriglyceridemia, low HDL cholesterol, hypertension, and impaired glucose metabolism [5]. Although closely connected with obesity, MetS was associated with an increased mortality, even in normal-weight individuals [6].

The etiology of obesity is complex and combines genetic, behavioral, environmental, psychological, social, and even cultural influences [7]. Twin, family, and adoption studies suggest that as much as 40% to 70% of the body weight index variation is explained by genetic factors [8,9]. Genetic influences on obesity may be divided into two distinct categories. The first one is monogenic obesity, which is typically early-onset and severe. It is caused by a mutation in a single gene, such as one encoding leptin, leptin receptor, or melanocortin receptor 4 [10,11]. The second, more frequent type is polygenic obesity, caused by multiple mutations or common population variants in several genes. It increases individual susceptibility to environmental factors that cause obesity and is also characterized by later onset and reduced severity [10].

Although many genes are known to influence polygenic obesity, polymorphisms of fat mass and the obesity-associated protein (*FTO*) gene were quickly recognized as highly influential regarding BMI and body adiposity [12,13]. FTO is a Fe (II)- and 2-oxoglutarate-dependent N6-methyladenosine demethylase, which removes methyl groups from N6-methyladenosine in mRNA and thusly reverses a common eukaryotic post-translational modification motif [14]. Single-nucleotide polymorphisms (SNPs) in *FTO* gene intron 1 have been shown to affect *FTO* expression [15,16], while FTO protein itself appears to influence adipogenesis by increasing the abundance of a short splice variant of RUNX1 translocation partner 1 (RUNX1T1) [17,18,19,20]. FTO controls the splicing of the adipogenic regulatory factor RUNX1T1 by regulating m6a. M6a affects several cellular processes, such as gene regulation and degradation, translation, or transport, and modulates alternative splicing and mRNA stability. An inverse correlation has been demonstrated between FTO and m6a, which is implicated in adipogenesis and demonstrates how FTO directly modulates obesity at the level of m6A [21]. Interestingly, SNPs of intron 1 of *FTO* have also been suggested to affect the expression of adjacent genes such as Iroquois homeobox 3 (IRX3), Iroquois homeobox 5 (IRX5), and RPGR-Interacting Protein 1-Like (RPGRIP1L) [22,23,24]. Another possible FTO role in obesity is the regulation of macronutrient intake due to the involvement of *FTO* expressed in the hypothalamus [25,26,27]. Although the overall impact of *FTO* gene polymorphisms on BMI and body composition was established, the impact and role of specific SNPs are less clear [12,28]. The occurrence of different polymorphic variants (SNPs) in the *FTO* gene may explain why unbalanced results in obesity have been obtained to date. Some reports suggest that specific haplotypes of the *FTO* gene influence rather than just particular SNPs complicating the issue further [21,29].

Thus, in this study, we aimed to determine the influence of polymorphisms in the *FTO* gene on the BMI and MetS components and assess the impact of genetic variants on the type of diet and lifestyles in a homogenous population of young Polish males [30].

## 2. Materials and Methods

### 2.1. Study Group

**Figure 1:**
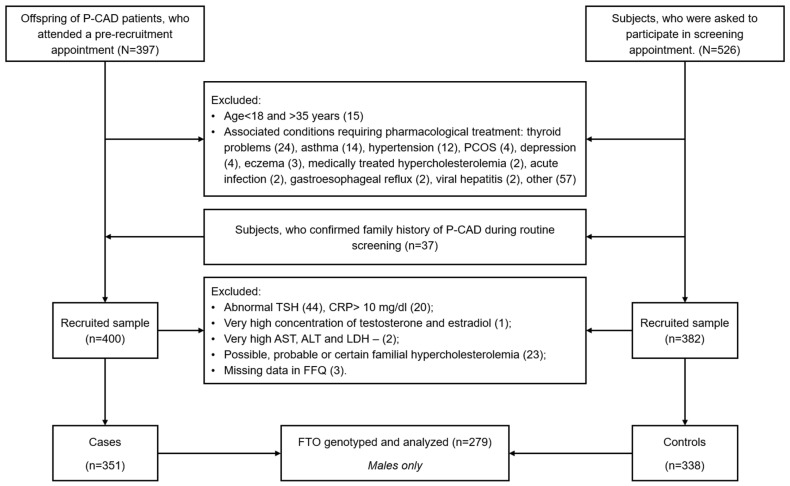
A flowchart of MAGNETIC study.

This study is an extension of the MAGNETIC (“Metabolic and Genetic Profiling of Young Adults with and without a Family History of Premature Coronary Heart Disease”) project. The study group consisted of randomly selected male participants recruited within the framework of the MAGNETIC project [31]. Briefly, the MAGNETIC project was a case-control study involving young (18–35 years) adults with (cases) and without (controls) a history of premature coronary artery disease (myocardial infarction, coronary artery bypass grafting, or percutaneous coronary intervention before the age of 55 in men and 65 in women) in first-degree relatives (Figure 1).

For the purposes of this study, only men were chosen because there was a higher percentage of obese people with MetS among men than women. None of the study participants was taking lipid-lowering, hypotensive, or anti-diabetic drugs.

The criteria for a MetS diagnosis were based on the Joint Interim Statement Criteria and were [5]:

- Waist circumference ≥80 cm in women and ≥94 cm in men;
- Fasting blood triglycerides > 1.7 mmol/L or treatment of hypertriglyceridemia;
- HDL-C < 1.3 mmol/L in women and <1.0 mmol/L in men or treatment of low HDL-c levels;
- Systolic blood pressure ≥ 130 mmHg or diastolic blood pressure ≥ 85 mm Hg or treatment for hypertension;
- Fasting blood glucose levels ≥ 5.6 mmol/L or treatment of DM.

At least three criteria had to be met for a MetS diagnosis. These criteria were chosen due to being widely used for MetS diagnostics.

### 2.2. Measurements of Anthropometric Parameters

During an initial visit at the Silesian Center for Heart Disease, trained examiners took measurements of the participants’ height, weight, waist, and hip circumference, as well as their systolic and diastolic blood pressure.

### 2.3. Laboratory Tests

Whole blood samples were collected from each participant 8–10 h after the last meal. Biochemical and immunoenzymatic tests were carried out in a hospital medical diagnostic laboratory. Patient serum measures were performed using a Cobas 6000 analyzer (Roche, Basel, Switzerland). Fibrinogen concentrations were determined using a BCS XP analyzer (Siemens Healthcare, Erlangen, Germany).

### 2.4. Diet Quality and Lifestyle

To collect an interview on eating habits and nutrition knowledge, the study participants completed two validated questionnaires: the FFQ-6 and KomPAN. The FFQ-6 covers a wide range of products (62 foodstuffs) that are usually consumed in Poland and was previously validated [32]. The KomPAN questionnaire is based on the frequency of consumption of 24 food items in the last year. The KomPAN questionnaire also includes questions about physical activity, smoking, and smoking status. Physical activity was categorized as low (sitting, screen time, reading, light housework, or walking 1–2 h a week), moderate (walking, cycling, moderate exercise, working at home or other light physical activity performed 2–3 h/week), or high (cycling, running, working at home or other sports activities requiring physical effort over 3 h/week) [33].

### 2.5. Genetic Analysis

DNA was isolated from the study participant’s whole blood samples, which were stored below −80 °C. Isolation procedures were performed according to the manufacturer’s instructions, using the automated extraction system MagCore^®^HF 16 and MagCore^®^GenomicDNA Whole Blood Kit (RBC Bioscience, New Taipei City, Taiwan). The purity and concentration of DNA were determined using a NanoDrop spectrophotometer (Thermo Scientific, Waltham, MA, USA). DNA samples were diluted to a 15 ng/µL concentration in molecular biology-grade water and stored below −30 °C until analysis.

The study sample was genotyped for five variants of the *FTO* gene: rs1421085, rs1121980, rs8050136, rs9939609, and rs9930506 (Table S1). This was achieved using commercially available pre-designed TaqMan SNP genotyping assays (ThermoFisher, Waltham, MA, USA). Each genotyping reaction was conducted in a volume of 20 µL using FastStart Essential DNA Probes Master (Roche, Basel, Switzerland) on the real-time PCR device LifeCycler 96 (Roche, Basel, Switzerland). To ensure the credibility of the results, the genotyping of 10% of the samples was reattempted, achieving 100% repeatability.

### 2.6. Statistical Analysis

Analysis was performed using R language in the Rstudio environment [34]. In all calculations, results with a *p* < 0.05 were considered statistically significant.

Data were presented as mean and standard deviation (SD) or as absolute (*n*) and relative (%) frequencies in the tables. The Kruskal–Wallis rank sum or Chi-squared test was used to evaluate group differences.

All norms for the anthropometric and biochemical parameters were based on the Joint Interim Statement criteria [5].

The fast, exact test proposed by Wigginton, Cutler, and Abecasis [35], implemented in the SNPassoc package (version 2.1-0) [36], was used to determine the genotype’s consistency with the Hardy–Weinberg equilibrium.

Dietary patterns (DPs) were obtained by principal component analysis (PCA) with normalized varimax rotation based on the FFQ-6 questionnaire. To create these DPs, the frequency of consuming 26 food groups per day was standardized to have a mean of 0 and a standard deviation of 1. We utilized PCA, selecting components to keep, which were based on their interpretability and eigenvalues (>1), along with identifying a breakpoint through the Scree test. The significance of each questionnaire item’s contribution to the identified DPs was assessed through factor loadings exceeding > |0.30|. The dietary patterns were named according to the variables with the highest loadings for each pattern. For each individual, a DP score reflecting their adherence to the DP was computed as the sum of the product of food frequency consumption and the factor loading for the 26 food groups. The subjects were categorized into three groups (bottom, middle, and upper tertile) based on the tertile distribution, representing the lowest, moderate, and highest adherence to the DP, respectively [37].

Furthermore, two diet quality scores, the pro-Healthy-Diet-Index (pHDI) and non-Healthy-Diet-Index (nHDI), were calculated based on the KomPAN questionnaires and previously published methodology [38].

## 3. Results

### 3.1. Study Group Characteristics

The studied group consisted of 279 young adult males aged 18–36 (Table 1). The MetS criteria were fulfilled for 21.5% of the patients. The average BMI was above the overweight threshold (>25 kg/m^2^); 18.28% of the study participants were obese, and 32.98% were overweight. Excessive waist circumference was noted in 31.54% of the patients. Hypertriglyceridemia was noted in 19.71% of the patients, while low blood HDL-C levels were found in 47.67% of the studied population. High blood pressure was found in 57.71% of the studied group. Elevated fasting glucose was present in 17.92% of the studied persons, but none of the participants was diagnosed with DM. All patients were of Caucasian descent and were residents of Upper Silesia.

### 3.2. FTO SNP

All SNPs were located in the first intron of the *FTO* gene in a region spanning 29,511 bp. They were located between 4252 bp and 9938 bp distance from each other (Table S1). All genotypes of the five analyzed SNPs (rs1421085, rs1121980, rs8050136, rs9939609, and rs9930506) were in Hardy–Weinberg equilibrium. The allele frequencies did not differ significantly based on the BMI category, waist/hip ratio (WHR), HbA1c presence of MetS components, or a family history of T1D or T2D. The genotype frequencies of all tested polymorphisms are presented in Table S2.

During further analysis, seven haplotypes of the analyzed SNPs were identified. The respective alleles in each haplotype are arranged in the following order of SNPs: rs1421085, rs1121980, rs8050136, rs9939609, and rs9930506. The two most common alleles that were found in 95% of the participants were TGCTA (all protective alleles) and CAAAG (all risk alleles) (Table S3). Next, 12 diplotypes were identified (Table S4). In 89% of the participants, the diplotypes consisted of just the two most common haplotypes. According to the nomenclature proposed by Kolackov et al., a diplotype consisting of a pair of TGCTA haplotypes was termed a protective diplotype. The Diplotype consisting of a pair of CAAAG haplotypes was named risk diplotype 2, while the diplotype consisting of one TGCTA and one CAAAG haplotype was named risk diplotype 1 [29]. The protective diplotype was identified in 65 participants, risk diplotype 1 was found in 128 participants, while risk diplotype 2 was identified in 55 participants. Next, the levels of each MetS component in the participants of every identified diplotype were compared (Table 2 and Table S6). No significant differences were detected (*p* > 0.05).

### 3.3. Dietary Patterns, Dietary Quality, and Physical Activity

PCA was used to identify two dietary patterns (DPs), explaining a cumulative 24% of the variation in the analyzed variables (Table S5). The first “Prudent DP” was characterized by a frequent intake of milk, fermented milk drinks, and curd cheese (0.61), whole grain products (0.67), vegetables (0.67), fish (0.60), fruits (0.54), nuts and seeds (0.53), eggs and egg dishes (0.42), white meat (0.45), and legumes (0.45). The second, “Western DP” was characterized by more frequent consumption of sugar (0.51), refined grain products (0.51), processed meats (0.67), potatoes (0.61), sweets and snacks (0.49), animal fats (0.49), other edible fats (0.46), and sweetened beverages and energy drinks (0.45). When the three most common *FTO* diplotypes were compared regarding the DPs, knowledge of nutrition, or physical activity, no significant differences were found (Table 3); only “Risk diplotype 2” was characterized by a lower pHDI but this was not statistically significant (*p* > 0.05).

## 4. Discussion

In the age of the obesity epidemic, it is important to determine the causes of this phenomenon to enable the development of better ways of preventing and treating this disease.

Previously published analyses based on much larger studies and meta-analyses conducted on a broader population indicate that *FTO* genetic variants correlate with obesity. Some previously published analyses of the Polish population show that the influence of SNPs in the first intron of the *FTO* gene on obesity is modulated by age and gender. In our study, we investigated young adult men, while Sobalska-Kwapis et al. showed an association between obesity risk and SNPs in the *FTO* intron 1 in a group of 2747 men aged 45–50 years. Perhaps the lack of significance in our study was influenced by the properly functioning hormonal system in young men, which could compensate for the genetic predisposition to obesity [39,40]. Other studies concerning the Polish population have also assessed the impact of polymorphisms in the *FTO* gene on BMI and body mass. The study conducted by Piwonska et al. included a group of 3369 patients aged 20–74. In this study, the AA genotype of the rs9939609 variant was significantly correlated with body mass, BMI, waist circumference, and hip circumference [41]. Other analyses conducted by Luczynski et al. in a group of 968 Polish children aged 4–18 years indicated that AA homozygotes of the rs9939609 variant were characterized by increased body mass, BMI, waist circumference, arm circumference, and height [42]. A correlation between obesity and the rs9930506 variant was also demonstrated by Wrzosek et al. in a study of 442 Polish males [40]. Another study, which utilized sequencing of the chromosome 16 fragment encoding the *FTO* gene, has shown a strong correlation between rs9930506 polymorphism and obesity and being overweight in males and the rs1421085 variant and obesity in females. In the same study, a regression model, stratified by age and sex, has shown that allele A of rs9939609 was significantly more frequent in obese persons [43].

Many published analyses confirm the association of intronic polymorphisms of the *FTO* gene with obesity and BMI, but it is important to emphasize that there are also studies similar to those presented here that do not confirm the influence of *FTO* variants on the occurrence of obesity in the Polish population. In one such study, the rs9939609 polymorphism was analyzed in 1097 persons aged 30–80, but a correlation with BMI was found only among males [44]. During the 6-year follow–up, there was no significant impact of rs9939609 polymorphisms on body mass increase [45]. In another study, which was conducted by Kolačkov et al., there was no significant correlation between the *FTO* polymorphisms (rs1421085, rs1121980, rs9939609, and rs9930506) and BMI among persons with a BMI < 25, although there was a tendency for an increased hip circumference among women with haplotype TCGA [29]. This suggests that the influence of *FTO* polymorphisms on BMI in the Polish population may not be universal. We can, therefore, conclude that the relationship between *FTO* and obesity may be more complex than we thought. There may be several reasons why the population studied by our team did not show a clear relationship between the genetic variants in the *FTO* gene and BMI. It is possible that in the case of young Polish men, the genetic variants in the *FTO* gene examined by our team do not significantly affect the body weight of the subjects. Additionally, it should be emphasized that the observations from our study may result from a relatively small study group. To confirm this observation, analyses should be carried out on a much larger group of respondents.

The impact of genetic variants located within the first intron of the *FTO* gene on the occurrence and development of MetS, BMI, and obesity was also analyzed in other populations. In a study conducted by Guclu-Geyik et al., two *FTO* gene polymorphisms, rs9939609 and rs1421085, were evaluated in a population of adult Turks consisting of 1967 adult men and women. The mean age of women was 49.2 ± 11.8 years, and the mean age of men in this study was 50.1 ± 12.0 years. It was shown that both SNPs were strongly associated with MetS in men and with obesity in the studied group of women. Additionally, men carrying the rs1421085 C allele showed an increased risk of insulin resistance and a higher BMI, while this association was not as evident in women [46]. At the same time, it was observed that in some native populations, such as the indigenous population of Samoa and American Samoa, *FTO* variants, e.g., rs1421085, rs1121980, rs8050136, rs9939609, rs9939973, rs17817449, rs3751812, and rs7190492 do not have a statistically significant effect on the BMI [47]. In turn, analyses based on the indigenous Xavante population in Brazil showed a significant correlation between the rs9939609 polymorphism and the body obesity index and being overweight, and at the same time, showed no relationship with obesity, BMI, and waist circumference [48]. These observations indicate that it may be unlikely that the studied population and Polish population, in general, have a genetic make-up that completely negates the influence of *FTO* intron 1 polymorphisms on the BMI, but population-dependent genetic factors that modulate the impact of particular SNPs remain possible.

It should also be noted that physical activity is a factor that may modulate the impact of *FTO* polymorphisms on obesity. In a study involving 420 Brazilians aged 7–17, genotype AA of rs9939609 influenced the BMI and waist circumference only in physically inactive adolescents [49]. On the other hand, in a study of 1152 male Swedes, the same polymorphism showed no statistically significant correlation between the A alleles and BMI. It was observed that increased physical activity reduced the impact of allele A of rs9939609 on the BMI [50]. In our study, the population could be considered quite physically active since 35% of the studied patients reported vigorous physical activity, and a further 39% reported moderate physical activity; it is possible that this partially masks the influence of *FTO* variants on the BMI.

Additionally, in some populations, the impact of *FTO* polymorphisms on the BMI could be masked by dietary habits. In a study conducted by Harbron et al., a relationship of *FTO* polymorphisms (rs1421085 and rs17817449) with physical activity, dietary habits, and BMI was studied in Caucasian patients aged 25–40 with overweight or obesity. There was no direct correlation between the *FTO* polymorphisms and BMI, but two C alleles in rs1421085 increased the feelings of hunger and reduced dietary restraint and control [51]. A study enrolling over 6000 Koreans has shown that rs1121980 A allele carriers with a BMI ≥ 25 consumed more blue fish, organ meat, coffee, and coffee creamer. No such correlation was found in normal-weight males, although overweight and obese female rs1121980 A allele carriers consumed more sweets [52]. Although in our study *FTO* polymorphisms did not appear to affect dietary habits, it is nevertheless possible that the influence of *FTO* polymorphisms would be significant under a different diet than the one our patients had.

Another factor influencing the relationship between *FTO* gene polymorphisms and body mass is age. No correlation between the rs9939609 polymorphisms and BMI was seen in a study of 70-year-old individuals [53]. In another study, there was no impact of several *FTO* polymorphisms (rs9939609, rs8050136, rs1558902, and rs3751812) on the BMI in a group of 401 Han Chinese aged 14–18 [54]. In our study, the population was relatively young, with a mean age of 28.92. This may have affected the findings, although the patients were not elderly or underage, like in some studies. Moreover, some studies that included adolescents showed a correlation between *FTO* polymorphisms and BMI. For example, Kalantari et al. have reported that the GGC haplotype in rs9930506, rs9930501, and rs9932754 is associated with the BMI and related obesity rates in a group of 237 adolescent males aged 12–16 years [55].

In this study, there was also no significant correlation between the *FTO* polymorphisms and components of MetS other than obesity. This is consistent with the hypothesis proposed by other researchers that *FTO* influences lipid, glucose, and blood pressure levels indirectly, chiefly through its influence on body adiposity, body mass, and BMI. This is supported by a study conducted by Freathy et al., who analyzed data from 17,037 Caucasian patients. Upon adjusting for the BMI, there was no correlation between the rs9939609 alleles and metabolic syndrome components [56]. In turn, Doney et al., in a study of 4897 Scottish patients, found that the rs9939609 variants correlated with triglyceride and HDL-C levels, even after adjusting for obesity-related phenotypes [57].

The results presented by our research team in this publication have several strengths and weaknesses. The primary strengths of this work include the enrollment of patients from an understudied Polish population of young adult males and the availability of anthropometric parameters and laboratory test results, as well as physical activity and food intake data. The population was uniform regarding its ethnicity, and it must also be stated that the Polish population is uniform regarding its genetics [30]. Additionally, the study group was homogeneous in terms of age, with a mean age of 28.92 ± 4.28 of the examined males. Nevertheless, this work has some limitations. The analyzed population was rather small, so weak genetic influences could have been statistically insignificant, even if present. Moreover, the food intake and physical activity levels were self-reported, which may introduce some errors, even for validated questionnaires, although there is no ultimate gold standard for food questionnaires tailored to the Polish population. Additionally, the population likely had a genetically increased risk of premature coronary artery disease compared to the general population, and it makes it possible that some alleles of the SNPs of interest were enriched compared to the general population.

## 5. Conclusions

In this work, there is no statistically significant correlation between the BMI, MetS components, dietary habits, *FTO* polymorphisms, and diplotypes in a population of Polish young adult males.

## Figures and Tables

**Table 1:** Characteristics of included patients.

Variable	*N* *	Number of Patients (%) or Value (SD) ^†^
Age	279	28.92 (4.28)
Family History of P-CAD (%)	279	178/279 (64%)
Family History of T2DM (%)		99/279 (35%)
Current smoking (vs. past smoker or non-smoker)	279	72/279 (26%)
Physical activity level	279	
Low		73/279 (26%)
Moderate		108/279 (39%)
High		98/279 (35%)
SBP [mmHg]	270	132 (15)
DBP [mmHg]	270	81 (11)
BMI [kg/m^2^]	279	26.14 (4.38)
VAI	279	1.58 (2.65)
WC [m]	279	0.89 (0.08)
WHTR	279	0.50 (0.06)
TC [mmol/L]	279	5.12 (1.11)
HDL-C [mmol/L]	279	1.42 (0.37)
LDL-C [mmol/L]	279	3.26 (0.96)
TG [mmol/L]	279	1.42 (1.51)
Lp(a) [nmol/L]	279	42.49 (65.64)
apoA1 [g/L]	276	1.56 (0.25)
apoB [g/L]	277	1.02 (0.60)
Glucose [mmol/L]	279	5.15 (0.45)
HbA1c [%]	278	5.06 (0.25)
hsCRP [mg/dL]	279	1.33 (1.37)
Uric Acid [µmol/L]	279	349.24 (63.96)
Fibrinogen [mg/dL]	278	264.96 (56.89)

P-CAD—premature coronary artery disease; T2DM—diabetes mellitus type 2; SBP—systolic blood pressure; DBP—diastolic blood pressure; BMI—body mass index; VAI—visceral adiposity index; WC—waist circumference; WHTR—waist/hip ratio; TC—total cholesterol; HDL-C—high-density lipoprotein cholesterol; LDL-C—low-density lipoprotein cholesterol; TG—triglycerides; Lp(a)—lipoprotein(a); apoA1—apolipoprotein A1; apoB—apolipoprotein B; HbA1c—glycated hemoglobin; hsCRP—high-sensitivity C-reactive protein. * Full sample size is 279. Smaller *N* numbers indicate missing data. ^†^ Values are presented as mean (SD) or *n*/*N* (%).

**Table 2:** Analysis of components of metabolic syndrome in relation to *FTO* diplotype (three most common haplotype pairs).

Variable	Protective Diplotype *N* = 65 ^1^	“Risk” Diplotype 1 *N* = 129 ^1^	“Risk” Diplotype 2 *N* = 55 ^1^	*p*-Value ^2^
BMI [kg/m^2^]	25.86 (4.59)	26.46 (4.43)	26.07 (4.39)	0.53
WC [m]	0.89 (0.12)	0.90 (0.11)	0.90 (0.12)	0.55
TG [mmol/L]	1.26 (0.79)	1.39 (1.42)	1.45 (1.16)	0.72
HDL-C [mmol/L]	1.49 (0.40)	1.37 (0.33)	1.43 (0.39)	0.33
Glucose [mmol/L]	5.08 (0.34)	5.16 (0.47)	5.21 (0.50)	0.20
SBP [mmHg]	131.25 (12.41)	130.81 (14.86)	136.15 (15.33)	0.060
DBP [mmHg]	81.19 (9.25)	80.22 (11.33)	82.56 (12.89)	0.21

BMI—body mass index; SBP—systolic blood pressure; DBP—diastolic blood pressure; HDL-C—high-density lipoprotein cholesterol; TG—triglycerides; WC—waist circumference; ^1^ Mean (SD); ^2^ Kruskal–Wallis rank sum test; protective diplotype—TGCTA/TGCTA (rs1421085/rs1121980/rs8050136/rs9939609/rs9930506); risk diplotype 1—TGCTA/CAAAG (rs1421085/rs1121980/rs8050136/rs9939609/rs9930506); risk diplotype 2—CAAAG/CAAAG (rs1421085/rs1121980/rs8050136/rs9939609/rs9930506).

**Table 3:** Analysis of dietary patterns and physical activity in relation to *FTO* diplotypes (three most common haplotype pairs).

Variable	*N*	Protective Diplotype, *N* = 65 ^1^	Risk Diplotype 1, *N* = 128 ^1^	Risk Diplotype 2, *N* = 55 ^1^	*p*-Value ^2^
“Prudent” dietary pattern	248				0.61
Lowest adherence to DP		23/65 (35%)	44/128 (34%)	19/55 (35%)	
Moderate adherence to DP		20/65 (31%)	41/128 (32%)	23/55 (42%)	
Highest adherence to DP		22/65 (34%)	43/128 (34%)	13/55 (24%)	
“Western” dietary pattern	248				0.30
Lowest adherence to DP		21/65 (32%)	48/128 (38%)	14/55 (25%)	
Moderate adherence to DP		21/65 (32%)	46/128 (36%)	18/55 (33%)	
Highest adherence to DP		23/65 (35%)	34/128 (27%)	23/55 (42%)	
Physical activity	248				0.68
gentle		17/65 (26%)	33/128 (26%)	14/55 (25%)	
moderate		25/65 (38%)	55/128 (43%)	18/55 (33%)	
vigorous		23/65 (35%)	40/128 (31%)	23/55 (42%)	
nHDI (% points)	246	5.57 (2.42)	5.63 (2.18)	5.53 (2.74)	0.72
pHDI (% points)	247	6.87 (2.55)	6.26 (2.08)	7.18 (2.62)	0.087

DP—dietary patterns; pHDI—pro-Healthy-Diet-Index; nHDI—non-Healthy-Diet-Index; ^1^ Number of patients (%); ^2^ Kruskal–Wallis rank sum test or Chi-squared test; protective diplotype—TGCTA/TGCTA (rs1421085/rs1121980/rs8050136/rs9939609/rs9930506); risk diplotype 1—TGCTA/CAAAG (rs1421085/rs1121980/rs8050136/rs9939609/rs9930506; risk diplotype 2—CAAAG/CAAAG (rs1421085/rs1121980/rs8050136/rs9939609/rs9930506).

## Data Availability

The data presented in this study are available on request from the corresponding author. The data are not publicly available due to the planned preparation of subsequent publications based on the collected dataset (data may be publicly available after the end of the project, currently only upon reasonable request).

## Supplementary Materials

The following supporting information can be downloaded at: https://www.mdpi.com/article/10.3390/nu16111615/s1, Table S1: polymorphisms information; Table S2: Genotypes frequencies of *FTO* polymorphisms; Table S3: Haplotype frequencies; Table S4: Diplotype frequencies; Table S5: PCA-driven dietary patterns (DPs) identified in the study population (*n* = 278) by principal component analysis: data from FFQ-6 questionnaire; Table S6: Level HBA1c, BMI category, and familiar history of DM in relation to *FTO* diplotypes (three most common haplotype pairs).
