# Supplementary material for: The Impact of Haplotypes of the FTO Gene, Lifestyle, and Dietary Patterns on BMI and Metabolic Syndrome in Polish Young Adult Men
Source: Nutrients. 2024 May 25;16(11):1615. doi: 10.3390/nu16111615 (PMC11174437; doi:10.3390/nu16111615)
Supplement: Supplementary file 1 [file nutrients-16-01615-s001.zip › nutrients-2997169-supplementary.pdf]

Table S1. FTO polymorphisms information.

SNP	Risc	Alt	Position	HGVS	MAF	ClinVar Submission Accession	Risk Factor	GWAS	P-value
rs1421085	C	T	chr16:53800954	NG_012969.1:g.68080T>C	0.23	SCV000256632	Obesity	Obesity BMI (unit increase); Dietary macronutrient intake	1e-28; 7e-20; 1e-9
rs1121980	A	G	chr16:53809247	NG_012969.1:g.76373G>A	0.37	Not reported in ClinVar	Not reported in ClinVar	Obesity (early onset extreme); BMI (unit increase); circulating leptin levels (unit increase)	1e-7; 1e-15; 2e-15
rs8050136	A	C	chr16:53816275	NG_012969.1:g.83401C>A	0.32	Not reported in ClinVar	Not reported in ClinVar	Obesity (extreme); BMI (unit increase); Weight (percentage SD increase)	8e-10; 7e-17; 5e-36
rs9939609	A	T	chr16:53820527	NG_012969.1:g.87653T>A	0.34	Not reported in ClinVar	Not reported in ClinVar	BMI (unit increase adults)	3e-35
rs9930506	G	A	chr16:53830465	NG_012969.1:g.97591A>G	0.29	Not reported in ClinVar	Not reported in ClinVar	Obesity-related traits (SD decrease); BMI (unit increase)	3e-8; 4e-18

SNP - single nucleotide polymorphism
Risc - risk allele
Alt - alternative allele
Position - position in the genome according to Homo sapiens (human) genome assembly GRCh37 (hg19)
MAF - minor allele frequency (https://www.ensembl.org)
p-value, GWAS- https://varsome.com/gene/hg38/FTO 05-01-2024

Table S2. Genotypes frequencies of FTO polymorphisms.

SNP	Genotypes	N*	p†
**rs1421085**	T/T	71 / 279 (25%)	0.40
	C/T	147 / 279 (53%)	
	C/C	61 / 279 (22%)	
**rs9930506**	A/A	71 / 279 (25%)	0.90
	A/G	141 / 279 (51%)	
	G/G	67 / 279 (24%)	
**rs9939609**	T/T	77 / 279 (28%)	0.34
	A/T	147 / 279 (53%)	
	A/A	55 / 279 (20%)	
**rs8050136**	C/C	77 / 279 (28%)	0.34
	A/C	147 / 279 (53%)	
	A/A	55 / 279 (20%)	

**SNP**	**Genotypes**	**N***	**p†**
**rs1121980**	G/G	70 / 279 (25%)	0.63
	A/G	144 / 279 (52%)	
	A/A	65 / 279 (23%)	

SNP - single nucleotide polymorphism

*n / N (%), full sample size is 279.
† - p-value of chi-squared test for Hardy–Weinberg equilibrium

Table S3. Haplotype frequencies

**Haplotype***	**Frequency****
TGCTA	270 (48.39%)
CAAAG	257 (46.06%)
TGCTG	13 (2.33%)
CACTA	11 (1.97%)
TACTG	5 (0.89%)
CGCTA	1 (0.18%)
TACTA	1 (0.18%)

* Rs1421085 / Rs1121980 / Rs8050136 / Rs9939609 / Rs9930506
**n / N (%), the full sample size is 279

Table S4. Diplotype frequencies

Diplotype	Frequency*
TGCTA, CAAAG	129 / 279 (46%)
TGCTA, TGCTA	65 / 279 (23%)
CAAAG, CAAAG	55 / 279 (20%)
TGCTG, CAAAG	7 / 279 (2.5%)
TGCTA, CACTA	6 / 279 (2.2%)
CACTA, CAAAG	5 / 279 (1.8%)
TACTG, CAAAG	4 / 279 (1.4%)
TGCTA, TGCTG	4 / 279 (1.4%)
TACTA, CAAAG	1 / 279 (0.4%)
CGCTA, CAAAG	1 / 279 (0.4%)
TGCTA, TGCTG	1 / 279 (0.4%)
TGCTG, TGCTG	1 / 279 (0.4%)

*n / N (%), full sample size is 279

Table S5. PCA-driven dietary patterns (DPs) identified in the in the study population (n = 278) by principal component analysis: data from FFQ-6 questionnaire

	**Factor loadings**	
**Food items**	**Prudent DP**	**Western DP**
milk fermented milk drinks and curd cheese	0.61	
whole grain products	0.67	
vegetables	0.67	
fish	0.60	
fruits	0.54	

	Factor loadings	
nuts and seeds	0.53	
sugar		0.51
refined grain products		0.51
processed meats		0.67
potatoes		0.61
eggs and egg dishes	0.42	
honey		
sweets and snacks		0.49
sweetened milk products		
chesses		
breakfest cereals		
animal fats		0.49
red meats		0.34
venision		
vegetable oils		
other edible fats		0.46
white meat	0.45	
legumes	0.45	
juices		
sweetened beverages and energy drinks		0.45
alcohol		
Variance explained (%)	13.0	11.0

Factor loadings of >|0.30| are shown in the table, therefore three dietary items (out of 26) are not presented for simplicity. Sorted by loadings from 1st to 3rd factor. Total variance in dietary variables explained by three patterns is 24.0%

Table S6. Level HBA1c, BMI category and familiar history of DM in relation to FTO diplotypes (three most common haplotype pairs)

Variable	Protective diplotype N = 65[1]	“Risk” diplotype 1 N = 129[1]	“Risk” diplotype 2 N = 55[1]	p-value[2]
**HbA1c [%]**	5.02 (0.24)	5.07 (0.24)	5.11 (0.29)	0.14
**BMI category**				
Normal weight	36 / 65 (55%)	58 / 129 (45%)	26 / 55 (47%)	
Overweight	17 / 65 (26%)	44 / 129 (34%)	19 / 55 (35%)	
Obese	12 / 65 (18%)	27 / 129 (21%)	10 / 55 (18%)	
**DM**				0.97
Type 1	8 / 65 (12%)	16 / 129 (12%)	5 / 55 (9.1%)	
Type 2	25 / 65 (38%)	47 / 129 (36%)	22 / 55 (40%)	

[1] Mean (SD)
[2] Kruskal-Wallis rank sum test
protective diplotype - TGCTA / TGCTA (Rs1421085 / Rs1121980 / Rs8050136 / Rs9939609 / Rs9930506)
risk diplotype 1 -TGCTA / CAAAG (Rs1421085 / Rs1121980 / Rs8050136 / Rs9939609 / Rs9930506
risk diplotype 2 - CAAAG / CAAAG (Rs1421085 / Rs1121980 / Rs8050136 / Rs9939609 / Rs9930506)
Normal weight - BMI between 18.5 and 24.9
Overweight - BMI between 25 and 29.9
Obese - BMI 30 or over
HbA1c - Glycated hemoglobin
DM - diabetes mellitus
